# Genomic Breeding of Green Super Rice Varieties and Their Deployment in Asia and Africa

**DOI:** 10.1007/s00122-019-03516-9

**Published:** 2020-01-08

**Authors:** Sibin Yu, Jauhar Ali, Chaopu Zhang, Zhikang Li, Qifa Zhang

**Affiliations:** 1 National Key Laboratory of Crop Genetic Improvement, Huazhong Agricultural University, Wuhan, 430070 China; 2 International Rice Research Institute, DAPO Box 7777 Metro Manila, Philippines; 3 Institute of Crop Sciences, Chinese Academy of Agricultural Sciences, Beijing, China; 4 College of Agronomy, Anhui Agricultural University, Hefei, China

## Abstract

**Key message:**

The “Green Super Rice” (GSR) project aims to fundamentally transform crop production techniques and promote the development of green agriculture based on functional genomics and breeding of GSR varieties by whole-genome breeding platforms.

**Abstract:**

Rice (*Oryza sativa* L.) is one of the leading food crops of the world, and the safe production of rice plays a central role in ensuring food security. However, the conflicts between rice production and environmental resources are becoming increasingly acute. For this reason, scientists in China have proposed the concept of Green Super Rice for promoting resource-saving and environment-friendly rice production, while still achieving a yield increase and quality improvement. GSR is becoming one of the major goals for agricultural research and crop improvement worldwide, which aims to mine and use vital genes associated with superior agronomic traits such as high yield, good quality, nutrient efficiency, and resistance against insects and stresses; establish genomic breeding platforms to breed and apply GSR; and set up resource-saving and environment-friendly cultivation management systems. GSR has been introduced into eight African and eight Asian countries and has contributed significantly to rice cultivation and food security in these countries. This article mainly describes the GSR concept and recent research progress, as well as the significant achievements in GSR breeding and its application.

**Electronic supplementary material:**

The online version of this article (10.1007/s00122-019-03516-9) contains supplementary material, which is available to authorized users.

## Introduction

Rice (*Oryza sativa* L.) is one of the most important food crops and is the primary staple food for nearly half of the world’s population. It is expected that the world population will continue to grow and exceed nine billion by 2050, which demands a nearly 70% increase in food production (FAO 2013; http://faostat.fao.org/). Hence, increasing rice yield is critical to ensuring the world food security and living standards of everyone. The breeding and cultivation of semi-dwarf rice and hybrid rice varieties have contributed to two great leaps in rice productivity (Khush 2001). However, the breeding and wide adoption of many semi-dwarf, fertilizer-responsive/tolerant, and high-yielding varieties have also caused the overuses of chemical fertilizers, pesticides, and water resources (Hazell et al. 1986). Particularly in Asia, the overuse of pesticides has caused severe damage to ecological environments. The application of large amounts of nitrogenous fertilizers and the low fertilizer-use efficiency have led to problems of severe soil degradation and eutrophication of water bodies. The shortage, low fertilizer-use efficiency, and uneven distribution of water resources have caused a series of environmental problems such as the prevalence of drought (Zhang 2007). In addition, because of the frequent occurrences of extreme weather worldwide, the conflicts between agricultural production and environmental resources are becoming increasingly intense (Yorobe et al. 2016). Therefore, ensuring the food security and sustainable development of agriculture has become a key strategic concern worldwide.

Asia contributes significantly with 90% of global rice production and its consumption (Elert 2014). Hence, the conflicts between rice production and environmental resources are particularly acute. To enhance rice yields, farmers have gradually increased the application amounts of fertilizers to meet the demands of high-yielding varieties for more nitrogen (Ali et al. 2018). In recent decades, the average rice productivity in China rose from 2.1 t ha^−1^ in 1961 to 6.7 t ha^−1^ in 2013, which was accompanied with greatly increased application of nitrogen fertilizer from 8 to 35% of total amount of fertilizers used in the world (Wang and Peng 2017). The overuse of nitrogenous fertilizers and low fertilizer-use efficiency have caused large residual amounts of nitrogenous fertilizer to enter the soil and water bodies around farm lands, leading to severe environmental pollution (Peng et al. 2002; Ali et al. 2018). Also, the frequent occurrences of drought are another factor that hinders agricultural development, posing significant threats to world food security (Luo 2010). China suffers severely from water deficit, especially for agriculture, and is frequently hit by droughts. Since the 1990s, about 26 million hectares of its arable lands have been affected by drought every year, which directly led to a loss of 70 million tons in food crop production (Jing 2007). Meanwhile, the total irrigation water used for rice production accounts for nearly 70% of the total water amount used for agricultural production. This limited water resource can barely meet the demand for rice production in China (Zhang 2007). Hence, the breeding and cultivation of new types of varieties with superior resistances/tolerances to drought and pests, greatly improved water and nutrient (nitrogen and phosphorus) use efficiency, as well as high-yield potential and desirable grain quality have become a crucial goal of rice improvement to increase and/or stabilize rice productivity, alleviate the water shortage, protect the ecological environments, and ensure food security of China.

## The GSR project and its major research themes

Facing the increasingly severe resource shortage, environmental pollution, and degradation of ecological systems, Chinese scientists proposed the GSR project in 2005, which aims to develop new rice varieties with various green traits, including resistance to multiple insects and/or diseases, high use efficiency of fertilizers, water-saving, drought tolerance, and stress resistance on the basis of high grain yield and quality (Zhang 2007). Now, the GSR concept not only refers to new varieties with green traits but also represents the green “resource-saving and environment-friendly” conceptualization of crop breeding technology and “high-yielding, high-efficient, ecological, and safe” crop management systems (Zhang 2007; Wing et al. 2018). After the proposal of the project, it has been strongly supported by the Chinese Government and international funding programs. In 2009, the Bill and Melinda Gates Foundation funded the international cooperation project on “Green Super Rice for Resource-poor farmers of Africa and Asia” (OPP1130530). In 2010, the Ministry of Science and Technology of China granted the 863 Project, “Breeding and Development of Green Super Rice,” with extended funding up to 2018 (2014AA10A600).

The GSR project has five main focuses (Zhang et al. 2018): (1) development and improvement of the theoretical and technical systems for GSR breeding. These systems and technologies were established at the population, individual, trait, and genome levels, and the strategies to combine the genomes and green traits of various germplasm resources for breeding of the GSR varieties proposed; (2) establishment of whole-genome selection platforms based on the recent developments/findings in the rice functional genomics research worldwide, including whole-genome breeding databases for molecular-designed breeding and gene chips; (3) development of new germplasm resources by pyramiding of genes of green traits, including development of novel germplasms with improved resistances to multiple abiotic (primarily drought) and biotic stresses, high water and nutrient-use efficiencies, and high grain yield and quality; (4) breeding of new GSR cultivars (both inbred and hybrid) with various combinations of green traits, improved grain yields and quality; (5) high-yield cultivation and field management techniques for GSR. The techniques and criteria for assessing green traits were established. In addition, “resource-saving and environment-friendly” cultivation management systems were set up and GSR varieties were widely planted. According to the GSR concept, we tentatively define newly developed GSR cultivars for a specific target rice ecological area into one of the following 4 main types: (1) water-saving and drought-resistant (WDR) cultivars that have the same or better grain yield and quality as the current check varieties under the normal irrigated conditions but yield 30% or more under the water-deficit or drought conditions; (2) nutrient-use-efficient (NUE) cultivars that show the same or better grain yield and quality as the current check varieties but with 30% of less fertilization (nitrogen and/or phosphorus) application; (3) pest-resistant cultivars that have significantly enhanced pest resistance to one or more key pests (with a 30% or more reduction in pesticide application); and (4) stress (salt, alkalinity, cold, heat, etc.)-tolerant cultivars that have the same or better grain yield and quality as the current check varieties under the non-stress conditions but yield 30% or more than the checks under the stress conditions. In the program, GSR varieties to be developed may carry different combinations of the green traits depending on yield limiting factors in any specific environments.

## Genomic breeding for GSR

Based on the concept of Green Super Rice with less inputs, more production, and a better environment, several breeding strategies for developing GSR cultivars were formulated by integrating germplasm accessions, genomic resources, and molecular technology and breeding tools (Zhang 2007; Ali et al. 2018; Wing et al. 2018). Germplasms are essential materials for the genetic improvement and functional genomics research of crops and are strategic resources to support the sustainable development of GSR as well. With a long planting history, rice is rich in genetic/genomic diversity with huge numbers of rice germplasm collections, including both cultivated species and its closely related wild species maintained in gene banks worldwide (Wing et al. 2018). With the rapid advancement of DNA-sequencing and multi-omics technology, whole-genome analyses of gene variations and genome diversity of different types of rice germplasm resources have become an essential part of rice germplasm characterization and utilization. These abundant rice germplasm resources provide sufficient materials for further dissecting the genetic basis of complex traits, identifying novel genes and their functions for future rice molecular breeding by design (Xie et al. 2015; Wang et al. 2015b, 2018a).

**Fig. 1:**
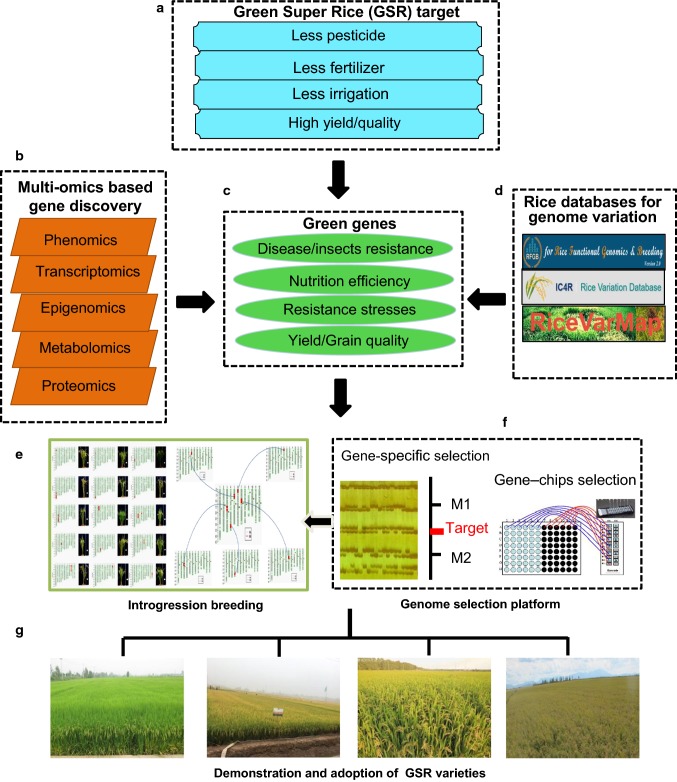
The target, strategy, and design for the development of Green Super Rice (GSR). **a** The goal of GSR was proposed to promote sustainable rice production with less inputs, while still achieving a yield increase and quality improvement. **b** Integration of multi-omics (phenomics, genomics, transcriptomics, epigenomics, metabolomics, and proteomics) to identify and understand green genes (such as high yield, good grain quality, resistance to stresses, nutrient-use efficiency). **c** A series of databases for rice genomic variations. **d** Schematic illustration of introgression lines, each containing a target gene and that could be combined in various ways to develop GSR. **e** The rice gene-chip and the gene-specific selection system. **f** Adoption of GSR varieties in various ecosystems

**Fig. 2:**
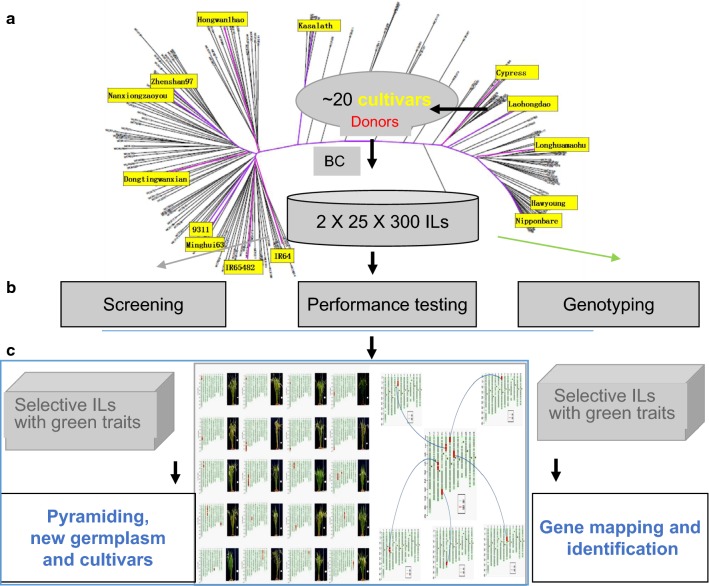
Massive backcrossing strategy for the development of GSR varieties: **a** Large-scale cross of diverse donors with elite parents and massive repeated backcrossing (BC) to one elite parent to generate near-isogenic lines or introgression lines (IL) with the desirable genes or traits. **b** Genotyping and phenotyping of the selective IL population to identify genetic variations for the target traits. **c** Elite lines carrying a single gene of interest may be combined to develop GSR cultivars

Based on the research advances in the rice functional genomics and genomic diversity, different genomics-assisted breeding strategies have been adopted for the development of the four major types of GSR cultivars (Wing et al. 2018). By combining multi-omics such as genomics, phenomics, epigenetics, metabolomics, proteomics, and transcriptomics, the desirable genes in the wild and cultivated rice species were mined and identified through large-scale and high-throughput phenotypic analyses on the re-sequenced rice germplasm (including wild species) accessions. A series of near-isogenic lines (NILs) or introgression lines (ILs) with the elite genetic backgrounds (widely planted major rice varieties and hybrid parents) that contain only small genomic segments (e.g., about 200 kb) of target genes were created through the whole-genome selection platform (Fig. 1). This genome selection platform comprising specifically designed gene chips (Yu et al. 2014; Chen et al. 2014) and selection systems simultaneous selecting any specific groups of target genes, non-target ones, and the entire genome background, which is expected to facilitate the accurate manipulation and improvement of targeted green traits (Wing et al. 2018). Another strategy involves an introgression breeding scheme in which large-scale crossing and massive repeated backcrossing to one (or more) elite parent are performed to generate NILs with the desirable traits or genes (Fig. 2). Such NIL populations are coupled with genotyping and massive phenotyping to identify genetic variation associated with critical green traits. Then, pre-GSR lines or GSR varieties were developed in a two-stage process. In the first stage, elite lines carrying a single gene of interest were developed and thoroughly evaluated for the green traits, which by themselves were useful as pre-breeding GSR lines. Second, the genes introduced into these lines would be combined in a designed way to develop cultivars with favorable traits. The introgression breeding approach has been used for developing GSR, and demonstrates being robust in several successful applications (Li and Ali 2017; Feng et al. 2018; Liang et al. 2018), because of its advantage with simultaneous improvement and genetic dissection of complex traits by introgressing one or more target genes through the genome selection system. This approach resulted in many GSR varieties and their adoption across Asia and Africa in the IR64, Huanghuazhan (HHZ), and Weed Tolerant Rice 1(WTR1) recipient backgrounds (Ali et al. 2018).

## Green genes for the breeding of new GSR varieties

Currently, more than 3000 genes affecting a wide range of phenotypes have been cloned and dissected in rice (up to 2018, www.ricedata.com; Li et al. 2018b; Wing et al. 2018). Of these cloned rice genes, those associated with resistance to biotic stresses (diseases and insect pests) and abiotic stresses (drought, salinity, flooding, low inputs), and traits of high nitrogen- and phosphorus-use efficiency, high yield, and good grain quality, are mostly “resource-saving and environment-friendly” and can be referred to as green genes. These green genes have been the targets for GSR breeding (Table 1).

**Table 1:** Representative genes related to green traits from 2014 to 2019

Green trait	Gene	Accession number	Chr.	Function causes	References
Grain yield/grain quality	*OsAAP6*	LOC_Os01g65670	1	Expression change by variations in promoter	Peng et al. 2014
	*GL2/GS2/OsGRF4*	LOC_Os02g47280	2	Expression change	Che et al. (2015), Duan et al. (2015) and Hu et al. (2015b)
	*GNP1*	LOC_Os03g63970	3	Expression change by variations in promoter	Wu et al. (2016)
	*lgy3*	LOC_Os03g11614	3	Protein structure	Liu et al. (2018a)
	*GL3.3/TGW3/qTGW3*	LOC_Os03g62500	3	Premature termination	Hu et al. (2018), Xia et al. (2018) and Ying et al. (2018)
	*Chalk5*	LOC_Os05g06480	5	Expression change by variations in promoter	Li et al. (2014)
	*GW5*	LOC_Os05g09520	5	Expression change by variations in promoter	Liu et al. (2017)
	*GW7/GL7*	LOC_Os07g41200	7	Expression change by variations in promoter	Wang et al. (2015a, c)
	*GLW7/OsSPL13*	LOC_Os07g32170	7	Expression change by variations in promoter	Si et al. (2016)
	*OsOTUB1*	LOC_Os08g42540	8	Expression change by variations in promoter	Wang et al. (2017b)
Disease/insect pest resistance	*Bsr*-*d1*	LOC_Os03g32230	3	Expression change by variations in promoter	Li et al. (2017)
	*Bph3*	LOC_Os04g12540	4	Amino acid substitution	Liu et al. (2015)
		Os04g0202350	4	Premature termination	
		LOC_Os04g12580	4	Amino acid substitution	
	*BPH6*	LOC_Os04g35210	4	Amino acid substitution and deletion	Guo et al. (2018)
	*PigmR*	LOC_Os06g17900	6	Amino acid substitution	Deng et al. (2017)
	*IPA1*	LOC_Os08g39890	8	Expression change by variations in promoter	Wang et al. (2018)
	*STV11*	LOC_Os11g30910	11	Amino acid substitution and deletion	Wang et al. (2014)
	*Xa10*	LOC_Os11g37620	11	Gene deletion	Tian et al. (2014)
	*BPH9*	LOC_Os12g37280	12	Amino acid substitution and deletion	Zhao et al. (2016)
Nutrient-use efficiency	*DEP1*	LOC_Os09g26999	9	Amino acid substitution	Sun et al. (2014)
	*NRT1.1B*	LOC_Os10g40600	10	Amino acid substitution	Hu et al. (2015a)
	*GRF4*	LOC_Os02g47280	2	Expression change	Li et al. (2018a)
Cold resistance	*LGS1*	LOC_Os02g47280	2	Expression change	Chen et al. (2019a, 2019b)
	*COLD1*	LOC_Os04g51180	4	Amino acid substitution	Ma et al. (2015)
	*CTB4a*	LOC_Os04g04330	4	Expression change by variations in promoter	Zhang et al. (2017a)
	*bZIP73*	LOC_Os09g29820	9	Amino acid substitution	Liu et al. (2018b)
	*HAN1*	LOC_Os11g29290	11	Expression change by variations in promoter	Mao et al. (2019)
Heat resistance	*OsTT1*	LOC_Os03g26970	3	Amino acid substitution	Li et al. (2015)

One important component of the GSR project was to resequence a core collection of 3024 rice germplasm accessions and phenotype the sequenced lines and GSR parents plus a large set of chromosomal segment substitution lines (CSSLs) for many green traits to discover and mine genes/QTLs associated with the green traits by genome-wide association analyses. As a result, a large number of loci associated with green traits were identified (Sun et al. 2015; Zhu et al. 2015; Lv et al. 2016; Qiu et al. 2017; Liang et al. 2018). These newly detected QTLs or the cloned genes provide abundant genetic resources for the development of new GSR varieties.

To facilitate more efficient and accurate integration of the genotyping technology in GSR breeding, a pedigree analysis system of rice varieties was built by taking advantage of high-throughput SNP chips. The system was used for genotypic identification of the pedigree and derivative pedigree materials of a superior rice variety, Huanghuazhan (HHZ) for GSR development. As a result, a total of 1113 conserved and traceable chromosomal regions were identified, including genes related to many important agronomic traits, such as *sd1* (controlling plant height), *Ehd4* (controlling heading date), *htd1* (controlling tiller height and dwarfing)*, SSIIa* (controlling soluble starch synthesis), *GS3* (controlling grain size)*, Amy3A* (controlling amylase), *Gn1a* (controlling grain number), and *TAC1* (controlling tillering angle) (Zhou et al. 2016a; Chen et al. 2017).

The sequence analysis of a large number of varieties resulted in the identification of the selected genomic regions during breeding and characterized two subpopulations (*I* and *II*) for the *Xian* (*indica*) subspecies, which have distinct geographic origins, resulting from separate breeding activities of China and the International Rice Research Institute during the early “Green Revolution.” In addition, about 200 genomic regions subjected to different selections among different *Xian* subpopulations were found (Xie et al. 2015). These regions cover some genes with known functions and related to green traits as well as many loci with unknown functions. These selected loci will provide important targets for the further improvement of rice.

Many cloned genes with disease resistance have been widely applied to the breeding and improvement of new GSR varieties (Jiang et al. 2016; Hu et al. 2016, 2017). These included introduction of *Pi2* (blast resistance gene) and *Xa23* (bacterial blight resistance gene) into the photo-thermo-sensitive genic male sterile (PTGMS) line Guangzhan63-4S, which significantly enhanced the resistance of newly developed two-line hybrid breeding lines to bacterial blight and blast (Jiang et al. 2015a). In addition, two genes, *Bph14* and *Bph15* conferring resistance to brown planthoppers were together introduced into a superior restorer line, Huahui 93. The resultant NILs carrying *Bph14* and *Bph15* showed much improved resistance to brown planthoppers (Wang et al. 2016). Through marker-assisted backcross breeding, new 9311 lines carrying different combinations of brown planthopper resistance genes/loci (*QBph3*, *QBph4*, *Bph6*, *Bph3*, *Bph9*, *Bph10*, *Bph14*, *Bph15*, *Bph17*, *Bph18, Bph20*, *Bph21,* and *Bph24)* were developed, which showed stronger brown planthopper resistance at the seedling stage (Xiao et al. 2016). Recently, a novel gene, *Bph38(t)* on the long arm of chromosome 1, was mapped to a small genomic region of 496.2 kb explaining the phenotypic variation of 35.9% in a backcross population derived from a cross of HHZ and Khazar (Balachiranjeevi et al. 2019).

Breeding of new GSR varieties with high nitrogen-use efficiency is a critical way to reduce the application of nitrogenous fertilizer in rice production and is one of the primary goals of our GSR breeding as well. Jewel et al. (2019a) reported a unique and systematic breeding approach through the selection of introgression lines with higher nutrient-use efficiency (NUE) through the early backcross breeding program. The selection of ILs was carried out for four consecutive seasons under different combinations of N, P, and K dosages of fertilizer. Five promising ILs (Nue-115, Nue-114, Nue-112, Nue-229, and Nue-230) were identified as having high grain yield and significantly improved NUE. These ILs provided valuable materials and information in rice breeding programs for high NUE. Quantitative trait loci (QTLs) related to NUE were identified earlier by several researchers (Liu et al. 2016; Zhou et al. 2017a, b; Feng et al. 2018). Recently, Jewel et al. (2019b) detected a total of 49 main-effect QTLs in six nutrient conditions. These QTLs explained phenotypic variation ranging from 20.3 to 34.7% and were located on all 12 chromosomes, except on chromosomes 7, 11, and 12. Among these QTLs, four hotspot QTLs were identified on chromosomes 3, 5, 9, and 11. Interestingly, novel QTLs for partial factor productivity (22 QTLs) and agronomic efficiency (four QTLs) were detected for –P and 75% of recommended N conditions. Several candidate genes were identified in these QTLs regions, and they were involved in nutrient uptake and transporting mechanisms. Mahender et al. (2019) identified a total of 19 QTLs associated with three favorable agronomic traits by using tunable genotyping-by-sequencing technology. Interestingly, two QTLs (*qLC*-*II_1* and *qLC*-*II_11)* were detected for chlorophyll content under zero percentage of N, P, and K fertilizer. Together, these QTL regions and candidate genes would be of great value for marker-assisted selection and pyramiding of multiple QTLs for improving NUE in rice.

Several genes related to high nitrogen-use efficiency have been cloned. These included *OsNRT1* (Lin et al. 2000), *OsDUR3* (Wang et al. 2012a), *OsPTR6* (Fan et al. 2014), *qNGR9/DEP1* (Sun et al. 2014), *NRT1.1B* (Hu et al. 2015a), *OsNRT2.3* (Fan et al. 2016), and *GRF4* (Li et al. 2018a). Among them, *NRT1.1B* appears to be the most promising one. The primary function of this gene is its transport activity of nitrate and is strongly induced by nitrate (Hu et al. 2015a; Zhang et al. 2019). This gene shows apparent differentiation between *Xian* and *Geng (japonica*), and *NRT1.1B* (the *Xian* allele) had undergone through strong artificial selection during domestication. The allelic variation of *NRT1.1B* was found to be responsible for the big difference in nitrogen-use efficiency between *Xian* and *Geng*. Thus, *NRT1.1B*-*Xian* has great application value in GSR breeding and production. The introduction of *NRT1.1B*-*Xian* into Xiushui 34, a late-maturing *Geng* variety in Zhejiang, China, resulted in the development of several new high-yielding lines with greatly improved NUE. Of these new lines, the best one yielded 10.2 t ha^−1^ under a moderate rate of nitrogen input (a reduction in nitrogen fertilizer per hectare from 180 to 100 kg in Hubei, China), exhibiting great potential for high nitrogen-use efficiency.

Abiotic stresses are the most important factors limiting rice productivity in many rice ecosystems. Among the various abiotic stresses, drought severely affects rice production and causes substantial yield losses in drought prone areas of rice (Hu and Xiong 2014). Thus, identification of loci for stress resistance and the development of GSR varieties with improved resistance to a single or multiple stresses are of great significance in solving the proble. In rice, several genes related to abiotic stress resistance have been cloned (Table 1). These included some loci or candidate genes associated with drought tolerance (Ren et al. 2005; Redillas et al. 2012; Uga et al. 2013; Zhu and Xiong 2013; Huang et al. 2014; Zhang et al. 2016). Several other genes are also of value for improving rice resistance to abiotic stresses. These included three cloned genes (*COLD1*, *bZIP73*, and *HAN1*) for cold tolerance at seedling stage (Ma et al. 2015; Liu et al. 2018b; Mao et al. 2019), *CTB4a* for cold tolerance at the booting stage (Zhang et al. 2017a) and *TT1* for high-temperature resistance (Li et al. 2015). Although many genes for stress resistance have been cloned, the most successful application of these cloned stress tolerance genes in rice breeding was *SUB1* for submergence tolerance. This gene has been successfully introgressed into several mega rice varieties which showed significantly improved yields under natural flooding (Ismail et al. 2013).

Besides the advantages of resistance to various diseases, insects, and stresses and high nitrogen-use efficiency, the newly developed GSR varieties are characterized by high quality and yield. Currently, several genes related to yield and grain quality have been cloned in rice (Xing and Zhang 2010; Wang et al. 2012b, 2015a). The elite allele of *IPA1* (*ipa1*-*2D*) for an ideotype is located in a tandem repeat sequence upstream of the *IPA1* gene, and variation in the gene structure would lead to a decrease in the methylation level in the promoter region of *IPA1*, resulting in increased expression of *IPA1*, which contributes to an ideotype and appropriate tiller number to promote yield (Wang and Wang 2017; Zhang et al. 2017b; Wang et al. 2018b). In addition, a gene that influences spikelet number can increase the number of rice grains to elevate yield by more than 5% (Wu et al. 2016). The grain quality of rice mainly comprises processing, appearance, and nutritional and cooking qualities, which directly or indirectly determine the value of rice. At present, several genes related to rice quality have been cloned (Sano et al. 1986; Gao et al. 2003; Bradbury et al. 2005). For example, *Chalk5* loosens the structure and affects the content of starch granules, causing chalkiness of rice grains (Li et al. 2014). Two other genes, *ssIIIa* and *wx*, can synergistically regulate rice grain quality, and molecular markers linked to these two genes have been designed for their application in breeding (Zhou et al. 2016b).

## Establishment of genome databases for rice breeding

During the implementation of the GSR project, several databases for rice genomic variations were established. These databases provide key platforms for gene functional research and whole-genome selection breeding for rice. For example, a core collection of 3024 rice accessions representing nearly 95% of the total genetic diversity of 780 thousand rice germplasm collection maintained in gene banks worldwide were re-sequenced and analyzed. As a result, a total of 42 million single nucleotide polymorphisms (SNPs) and more than 100 thousand structural variations (deletion, translocation, inversion, and duplications) were identified, and ~ 20 thousand new genes were discovered. The 3 K Rice Genome Project established the pan-genome of the Asian cultivated rice and revealed its population structure (Wang et al. 2018a). Based on the information on rice genomic variations, a new multi-functional and comprehensive rice functional genomics and breeding database (RFGB) has been established, which included a rice sequence polymorphism information retrieval system, an explorer visualization system of the genome, and data output system for specific genome regions (http://www.rmbreeding.cn/) (Sun et al. 2016).

Currently, the GSR project integrated the re-sequencing data of more than 6000 rice genomes worldwide, carried out haplotype analysis on the whole-genome structural variations, and investigated the selected genomic regions during the breeding process. As a result, a database of rice SNPs (http://variation.ic4r.org/) was created. A platform for the marker-assisted molecular breeding of GSR was established (http://47.92.174.110), which can help to compare genotypes and predict the performances of breeding materials. In addition, the database of rice genomic variations RiceVarMap v2.0 (http://ricevarmap.ncpgr.cn/v2/) was improved, integrating all data on genomic variations, annotation of the functional variations, phenotypes, and genome-wide association studies (Zhao et al. 2015). These databases provide abundant information resources for molecular design breeding to develop new GSR varieties with high yield, high quality, and general adaptability and could help to enhance the efficiency of GSR breeding (Fig. 1) and promote the transition from the conventional “experience-based breeding” to the highly accurate and efficiently designed breeding.

## Software for genomic selection breeding

Genomic selection (GS) refers to the establishment of a correlation between marker genotypes and phenotypes for predicting performances of breeding populations for unknown phenotypes based on the markers and phenotypes of related (smaller) reference populations. One of the advantages of GS is its higher efficiency relative to the traditional phenotypic selection methods practiced in the conventional breeding, as it requires phenotyping fewer hybrids, and is capable of predicting the phenotypes of hybrids that have not undergone field tests based on genotypic data (Xu et al. 2014; Spindel et al. 2016; Yang et al. 2017). The GSR project developed a GS software gblup.jar based on the new genomic best linear unbiased prediction (GBLUP-AD) that includes both the additive and dominance effects. This software can be used to predict the performances of multiple traits and environmental effect. The GBLUP-AD has been used to predict the phenotypes of rice hybrids based on NCII design, and the predictive ability was significantly improved (Wang et al. 2017a). Also, the project has designed the gblupdesign.jar software based on Java which is able to predict the founder parents with the highest breeding values and the ideal genotypes to be used for molecular design breeding.

## The platform of genomic selection breeding of rice

The platform of genomic selection breeding established based on the research findings of genomics, breeding chips, and high-throughput sequencing provided solid technical support for the genomics-assisted breeding strategies and rapid development of new GSR varieties (Fig. 1). This GS technology can be applied to commercial breeding to accelerate the progress of commercialization of breeding and promote the transition from the conventional breeding to more accurate and efficient genomic breeding.

The GSR project has also developed three high-throughput breeding chips of rice based on Illumina’s Infinium technology, which constitute a GS technical system in our GSR breeding efforts. The three breeding chips include a RICE6K chip (Yu et al. 2014), a RICE60K chip (Chen et al. 2014), and a RICE90K chip, which comprise 4473, 43,386, and 85,000 high-quality markers, respectively. Based on the Hiseq 4000, × Ten, and BGISEQ-500 sequencing platforms, a high-throughput and low-cost whole-genome selection breeding platform that is not restricted by any specific reference genomes was set up. This platform can be used for the construction of genetic linkage maps, genetic diversity analysis, variety identification, and gene/QTL mapping. Currently, the platform has been widely applied to the analysis of genetic diversity of rice germplasm, genome-wide association studies, and molecular breeding activities (Yu et al. 2016; Qiu et al. 2018; Zhang et al. 2018). For example, the whole-genome breeding technology has been applied toward the improvement of biotic resistance of elite rice cultivars that have been widely grown as founder parents. The perfect introduction of resistance genes for rice blast and brown planthopper significantly enhanced the disease/insect resistance of the parents without altering the overall agronomic traits. The introduction of multiple blast resistance genes into a single parent or variety has contributed to the generation of NILs with a highly consistent genetic background (Wing et al. 2018; Zhang et al. 2018). The combination of various NILs with different blast resistance genes resulted in the development of varieties with consistent agronomic traits and high and stable resistance. In addition, the *S5*-n and/or *f5*-*n* from the wide compatibility variety Dular were introduced into the restorer line 9311, and the rapid detection of its background with RICE6K revealed a background recovery rate as high as 99.4%, resulting in the generation of improved 9311 with wide compatibility (Mi et al. 2016).

## Development of new pre-breeding GSR lines with pyramided green genes

Based on the information on cloned green genes and loci, large-scale cross and backcross breeding was conducted to generate IL populations and lines abundant in green traits with wild rice, core germplasm, and specific local varieties as the donors (Fig. 2). As a result, a large number of restorer lines, male sterile lines, and new pre-breeding lines with the advantages of disease/insect resistance, weed-competitive ability, high nitrogen- and phosphorus-use efficiency, water-saving, drought tolerance, and high yield and quality were bred (Jiang et al. 2015b, 2016; Xiao et al. 2016; Wang et al. 2016; Dimaano et al. 2017). Meanwhile, these lines were screened and identified for their resistance to drought, low phosphorus, low nitrogen, weed-competitive ability, blast, bacterial blight, rice false smut, and rice planthopper, creating a batch of new germplasm with multiple green traits such as multi-resistance, high nutrient-use efficiency, water-saving, drought tolerance, and high yield and quality. For example, the superior *indica* varieties HHZ and restorer line 9311, which are widely planted in central and southern China and later identified as highly adaptable to Asian and African target locations, were used as the recipients to breed a series of disease-/insect-resistant NILs with multiple brown planthopper resistance genes (such as *Bph1*, *Bph14*, and *Bph15*) and disease resistance genes (such as *Pi2*, *Pi9*, and *Pikm*). The dominant genic male sterile line JiafuzhanS was used as a facilitator for outcrossing to generate new lines with several disease resistance genes (such as *Pi1*, *Pi2*, *Xa21,* and *Xa23*). Also, two-line hybrid lines Y58S, Hua1017S, and Hua1037S harboring various disease/insect resistance genes (such as *Pi2*, *Pi9*, *Pikm*, *Bph3*, *Bph14*, *Bph32*, *Xa7*, *Xa21,* and *Xa23*) and elite genes of aroma were generated, and exhibited excellent application potential. These research findings will provide abundant necessary materials for the use of important agronomic traits and crop improvement, and are of guiding significance to the molecular design breeding of NILs with the target traits.

Globally, more than USD 100 billion are spent annually for weed control of the crops (Appleby et al. 2001). Therefore, breeding of weed-competitive (WC) rice varieties is a critical solution to reduce tillage operations and decrease hand weeding and herbicide inputs in the direct-seeded rice system. In this regard, the drought pyramiding GSR variety IR83140-B-11-B performed well in partial weed control plots, yielding 2850 and 4610 kg ha^−1^ in the wet and dry seasons at the International Rice Research Institute (IRRI), respectively (Chauhan et al. 2015). At an early stage of the crop, the trials showed that grain yield in different GSR genotypes was positively correlated with leaf area. We initiated the systematic breeding of rice varieties with weed-competitive ability by standardizing the phenotypic screening protocol to identify the weed-competitive traits related to early seed germination, early seedling vigor, and weed-competitive components. The breeding materials were developed from four early-generation backcross populations derived from one common recipient parent, WTR-1, and four different donors, Y134, Zhong 143, Khazar, and Cheng Hui-448. These ILs were evaluated in three rounds of selection in upland weed-free, upland-weedy, and lowland-weedy conditions. Five ILs (G-6-L2-WL-3, G-6-RF6-WL-3, G-6-L15-WU-1, G-6-Y16-WL-2, and G-6-L6-WU-3) were found to be promising ILs in lowland-weedy conditions, whereas four ILs (G-6-Y7-WL-3, G-6-Y6-WU-3, G-6-Y3-WL-3, and G-6-Y8-WU-1) were found to have the highest grain yield under upland-weedy conditions (Dimaano et al. 2017). For the molecular genetics of weed-competitive rice cultivars, a total of 44 QTLs were mapped on 12 chromosomes, except on chromosomes 4 and 8, by using 677 high-quality SNP markers. Interestingly, 29 novel genetic loci were associated with early seed germination and early seedling vigor traits on chromosomes 1, 3, 5, 6, 7, 10, 11, and 12. The hotspot regions of chromosomes 11 and 12 were associated with multiple traits (Dimaano et al. 2019, unpublished). Many of these QTLs were co-localized with previous reported QTLs, which were related to germination rate, germination index, germination percentage, and germination time in different genetic backgrounds of mapping populations (Mahender et al. 2015).

## Demonstration and application of GSR varieties in China

Based on the latest findings in genomics and bioinformatics research, oriented transfer and pyramiding of the favorable genes associated with rice yield, quality, disease/insect resistance, drought tolerance, and nutrient-use efficiency were carried out by using different techniques such as pedigree breeding, backcross breeding, combining ability breeding, marker-assisted selection, and whole-genome selection breeding (Fig. 1), resulting in the generation of new materials with multiple elite genes and GSR varieties with various desirable traits.

As of the end of 2018, a total of 66 new varieties developed by the GSR project had been registered in China (Table S1). The cumulative planting area of these cultivars over five main rice-growing regions exceeded 6.67 million hectares from 2014 to 2018, laying a solid foundation for promoting the sustainable development of rice production. For instance, in a two-year experiment on nitrogen fertilizer reduction, the partial factor productivity of applied N of hybrid variety Huiliangyou 630 increased by 10.2% compared with that of the control; in addition, this variety is characterized by high quality and resistance to blast and bacterial blight. Overall, it has reached the standards of a GSR variety (10% increases in the use efficiency of nitrogen fertilizer, phosphorus fertilizer, and water, respectively, and with moderate resistance to one or more main diseases or insects). The cumulative planting area of Huiliangyou 630 reached 210 thousand hectares during 2015–2018. Under the reduction of 30% in nitrogen fertilizer, the partial factor productivity of applied N of Jingliangyouhuazhan in two years increased by 23.2% relative to that of the control. This variety also has the characteristics of disease resistance, high nitrogen-use efficiency, low cadmium accumulation, high yield and quality, and wide adaptability. The cumulative planting area of Jingliangyouhuazhan reached 530 thousand hectares during 2016–2018, increasing rice yield by 320 thousand tons and decreasing the input of pesticide and fertilizer by 160 million yuan (about USD 22.86 million). Owing to its high grain quality and multiple resistances, the variety Wushansimiao has been planted on a cumulative area of 730 thousand hectares in southern China. One new variety, Wushansizhan, which was derived from Wushansimiao, is characterized by the advantages of high resistance to blast; moderate resistance to bacterial blight, lodging, and cold; and high grain quality.

## International popularization and application of GSR varieties

**Fig. 3:**
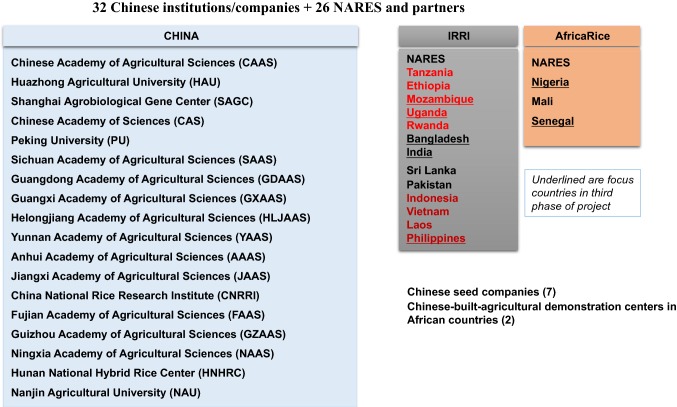
The GSR project participants in China, Asia, and Africa in collaboration with IRRI and AfricaRice

The demonstration and popularization of new GSR varieties have strongly promoted the development of superior rice production in the target regions. GSR materials were systematically introduced to 16 African and Asian countries with IRRI and AfricaRice for helping in the adaptation testing, breeding, and capacity building of local national agricultural research and extension system (NARES) partners. The GSR project involved 32 Chinese institutions, universities, academies, and seed companies partnering in the massive development and deployment of GSR products (Fig. 3). This led to the release of 59 GSR varieties that were adapted to the rice production areas of Africa and Asia (Table S2). Other 97 GSR cultivars were identified as promising and entered into national cooperative yield trials for their release in different target countries (Table S3). Among the 59 GSR varieties, HHZ was released in Mozambique, Indonesia, and India, demonstrating its wide-adaptation features. Further, HHZ provided an excellent base recipient parent for introgression breeding that led to the identification and release of 15 varieties with multiple stress tolerance (with GSR IR1 prefix) across different target countries in Asia and Africa. Interestingly, GSR variety HHZ was released in Punjab State in India as PR126 in 2017, which was of short duration (123 days) and yielded on a par with a dominant long-duration variety (Pusa 44), fitting well in the rice-potato cropping system. The average productivity per day over a hectare of PR126 was 61 kg vis-à-vis 46.9 kg of Pusa 44. Due to the earliness of PR126, Punjab saved on irrigation water and fertilizer while keeping the same yield levels of Pusa 44. PR126 is fast replacing predominant variety Pusa 44, especially in the rice-potato cropping belt, with a current adoption area of 31.6% (Veettil et al. 2019). This variety alone has created enormous socioeconomic and environmental impacts and is currently being studied. We have now created several hundred introgression lines with multiple abiotic stress tolerance in an HHZ background derived from different donors that outperform the recipient parent in grain yield and maturity. Soon, these lines may replace HHZ to create more significant socioeconomic impacts. Among them, 74 promising derivatives (with GSR IR1 prefix) in an HHZ background are being evaluated in national cooperative trials in India, Bangladesh, Pakistan, Indonesia, the Philippines, Mozambique, and Uganda (Table S3).

An excellent socioeconomic performance has been achieved by the popularization of GSR varieties in the target African and Asian countries. Socioeconomic assessment of some GSR varieties bred for rainfed and irrigated lowland environments in the Philippines and Bangladesh showed that GSR varieties contribute significantly to the yield and net income of farmers (Ali et al. 2012; Yorobe et al. 2016). Compared with conventional rice, GSR varieties showed significantly enhanced yield and economic output (by 0.89–1.83 tons and USD 230.90 per hectare, respectively) and disease/insect resistance (Yorobe et al. 2016). Currently, 59 varieties have passed regional tests and variety approval in countries in South Asia (Bangladesh, India, Pakistan, and Sri Lanka), Southeast Asia (Indonesia, Laos, Cambodia, the Philippines, and Vietnam), and Africa (Mozambique, Uganda, Rwanda, Nigeria, Senegal, and Mali) (Table S3). The cumulative area of the demonstration and popularization of GSR varieties in African, South Asian, and Southeast Asian countries has reached about 2.34 million hectares, which marked significant contributions to rice production and food security in these countries (Wang et al. 2018c).

## Identification criteria of GSR varieties and highly efficient cultivation systems

The breeding of superior varieties is a genetic approach, while improved cultivation techniques are non-genetic approaches to achieve higher grain yield and higher efficiency. The GSR project has established a complete assessment system for evaluating the green traits of GSR varieties and clarifying the related mechanisms. The establishment of a cultivation and field management system for GSR and a comprehensive prevention and control system for the primary diseases and insects provides essential support for the promotion of resource-use efficiency based on higher grain yield and stability, and for the environment-friendly and sustainable development of rice production.

The GSR project also successfully developed a series of key cultivation techniques for GSR varieties, such as site-specific nutrient management, precise water-saving irrigation technology, straw incorporation, and direct seeding (Wang and Peng 2017; Zhou et al. 2017a, b). During 2014–2018, the cumulative application area of GSR cultivation reached 3.48 million hectares (Zhang et al. 2018), thereby reducing the cost of fertilizer, pesticide, and water by 12.34 billion yuan (about USD 1.76 billion) in China alone.

The component “High-Yielding and Efficient Cultivation Technology of Ratooned Rice with Main Crop Harvested Mechanically” of the GSR project integrates ratoon rice varieties of high quality and yield and a series of key cultivation techniques. This project has implemented the demonstration and application of ratooned rice in large areas of Hubei Province, China, with a demonstration area of nearly 270 thousand hectares. In Hunan Province, a high-efficiency and high-yield cultivation technical system has been assembled, which is characterized by resistance to cold, high temperature, lodging, and banded sclerotial blight, and enhanced seedling regeneration ability. During 2014–2018, the cumulative application area of GSR cultivation was 110 thousand hectares. In Guangdong Province, the demonstration and application of “water- and fertilizer-saving” cultivation were conducted. During 2014–2016, the cumulative application area of this cultivation technique in this province reached 2.67 million hectares, reducing the cost of fertilizer and pesticide by 1.97 billion yuan (about USD 28 million), and increasing rice grain yield by 1.84 million tons and income by 7.31 billion yuan (about USD 1.04 billion).

## Prospects of GSR development

The world population is expected to reach nine billion in 2050. The rapid increase in population demands corresponding increases in rice production in a sustainable way, which will be a great challenge to global rice breeders in future decades. GSR is therefore a vital concept proposed to meet this challenge since GSR varieties can maintain stable and higher yield with less inputs, and have stronger resistance and recoverability features when facing the frequent occurrence of extreme stresses caused by climate changes. The practice in the past decade has demonstrated that the combination of GSR varieties and corresponding improved cultivation techniques can contribute to more stable and higher yields, and at the same time reduce the application of pesticide and fertilizer by more than 30%, as well as irrigation water by at least 30% in irrigated rice production areas. GSR may become a vital pattern to lead the green development of agriculture, and its goals and whole-genome breeding strategies may provide patterns or set examples for the development of other crops. The implementation of the GSR project has led to and promoted the transition of breeding goals and production modes of crops in China, and across the world (Wing et al. 2018).

The strategies to breed GSR varieties using multiple-omics not only greatly promote breeding accuracy and efficiency but also accelerate the commercialization and all-around transition and upgrading of crop breeding. By combining the abundant germplasm accessions, functional genomics, and molecular breeding with whole-genome selection, a large number of new varieties and accessions were used in the pyramiding of elite genes in pedigree breeding and backcross breeding. These varieties and accessions harbor various elite genes associated with high yield, superior grain quality, disease/insect resistance, drought tolerance, and higher nutrient efficiency. Genome-editing technology is becoming a key technology for genomic breeding owing to its advantages of high efficiency, low cost, and safety. For example, CRISPR/Cas technology provides efficient and versatile tools for efficient targeted modification of the genes of agricultural importance in crops and precision crop breeding (Chen et al. 2019a). Compared with conventional breeding, it greatly promotes the efficiency of pyramiding elite genes, and can create more abundant genetic resources with high yield, superior grain quality, and tolerance to various stresses.

During the demonstration and popularization of GSR varieties, technical training programs have conveyed the green concept and cultivation techniques of high quality, high yield, and efficiency to farmers, which significantly improves the technical quality of the workers or farmers in these areas. Also, the integration and innovation of green varieties and cultivation techniques have optimized rice production techniques, enhanced per unit area yield, and increased the income of farmers. An international network for cooperation involving the IRRI and AfricaRice, along with NARES partners in Africa and Asia, was established. In the short term, more and better GSR varieties with high adaptability to climate changes should be bred, alongside the development of corresponding green yield- and efficiency-promoting cultivation techniques. These goals require active cooperation among various fields of breeding, agronomy, agricultural machinery, and agricultural economics, with the sole purpose of increasing the income of small farm households in the target countries, ensuring food security, and promoting the sustainable development of global agriculture.

## Electronic supplementary material

Below is the link to the electronic supplementary material.

Supplementary material 1 (XLSX 23 kb)
