# Correction to: Genomic Breeding of Green Super Rice Varieties and Their Deployment in Asia and Africa

**DOI:** 10.1007/s00122-020-03550-y

**Published:** 2020-02-19

**Authors:** Sibin Yu, Jauhar Ali, Chaopu Zhang, Zhikang Li, Qifa Zhang

**Affiliations:** 1 National Key Laboratory of Crop Genetic Improvement, Huazhong Agricultural University, Wuhan, 430070 China; 2 International Rice Research Institute, DAPO Box 7777 Metro Manila, Philippines; 3 Institute of Crop Sciences, Chinese Academy of Agricultural Sciences, Beijing, China; 4 College of Agronomy, Anhui Agricultural University, Hefei, China

## Correction to: Theoretical and Applied Genetics 10.1007/s00122-019-03516-9

The article Genomic Breeding of Green Super Rice Varieties and Their Deployment in Asia and Africa, written by Sibin Yu, Jauhar Ali, Chaopu Zhang, Zhikang Li and Qifa Zhang, was originally published electronically on the publisher’s internet portal on 8th January 20120 without open access. With the author(s)’ decision to opt for Open Choice the copyright of the article changed on 28th January 2020 to © The Author(s) 2020 and the article is forthwith distributed under a Creative Commons Attribution 4.0 International License (https://creativecommons.org/licenses/by/4.0/), which permits use, sharing, adaptation, distribution and reproduction in any medium or format, as long as you give appropriate credit to the original author(s) and the source, provide a link to the Creative Commons licence, and indicate if changes were made.

The original article has been updated.

